# Beta-lactam antibiotic dosing during continuous renal replacement therapy: how can we optimize therapy?

**DOI:** 10.1186/cc13945

**Published:** 2014-06-26

**Authors:** Jan J De Waele, Mieke Carlier

**Affiliations:** 1 Department of Critical Care Medicine, Ghent University Hospital, De Pintelaan 185, 9000 Ghent, Belgium; 2 Department of Clinical Chemistry, Immunology and Microbiology, Ghent University Hospital, De Pintelaan 185, 9000 Ghent, Belgium

## Abstract

Correct antibiotic treatment is of utmost importance to treat infections in critically ill patients, not only in terms of spectrum and timing but also in terms of dosing. However, this is a real challenge for the clinician because the pathophysiology (such as shock, augmented renal clearance, and multiple organ dysfunction) has a major impact on the pharmacokinetics of hydrophilic antibiotics. The presence of extra-corporal circuits, such as continuous renal replacement therapy, may further complicate this difficult exercise. Standard dosing may result in inadequate concentrations, but unadjusted dosing regimens may lead to toxicity. Recent studies confirm the variability in concentrations, and the wide variation in dialysis techniques used certainly contributes to these findings. Well-designed clinical studies are needed to provide the data from which robust dosing guidance can be developed. In the meantime, non-adjusted dosing in the first 1 to 2 days of antibiotic therapy during continuous renal replacement therapy followed by dose reduction later on seems to be a prudent approach.

## 

This commentary discusses the findings of Beumier and colleagues [1] about beta-lactam concentrations during continuous renal replacement therapy (CRRT). In recent years, pharmacokinetics (PK) of antibiotics in critically ill patients has been studied intensely. Standard antibiotic dosing apparently results in highly variable antibiotic concentrations in the blood and tissues. A recent multicenter point prevalence study found that 20% of the patients are underdosed (when aiming for the most conservative target) and could link antibiotic underexposure to worse clinical outcome [2]. The causes for the variability in effective antibiotic concentration are multiple, but increased volume of distribution and enhanced elimination from the circulation, mostly via the kidneys in patients suffering from augmented renal clearance, are the most important contributors.

But also when the kidneys fail, antibiotic dosing can be challenging. When kidney function deteriorates, standard dosing may lead to accumulation of a drug and therefore antibiotic doses are often adapted to the kidney function. Also, when renal replacement therapy (RRT) is indicated, antibiotic doses are often decreased.

RRT does, however, affect antibiotic concentrations as antibiotics are also eliminated from the circulation. The rate at which this occurs is affected by the physicochemical characteristics of the drug and the type and intensity of RRT used, among other factors. Antibiotics with a small volume of distribution and low protein binding will be affected more. It can be expected that CRRT techniques are more efficient in removing antibiotics from the blood.

Several studies have addressed this issue in recent years. Seyler and colleagues [3] found that patients treated with CRRT had overall low target attainment in the early phase of therapy when recommended doses are used and when four times the minimum inhibitory concentration (MIC) of the least susceptible microorganism is aimed for [3]. Later during therapy, concentrations were higher for meropenem, piperacillin, and ceftazidime - in all patients, these drugs were administered as intermittent infusion. Bauer and colleagues [4] found that there was an important interindividual variability in piperacillin concentrations and that CRRT dose was the only factor associated with piperacillin elimination. This variability was confirmed by Roberts and colleagues [5] in a similar patient cohort, with both under- and overdosing occurring in about 10% of the patients, depending on the PK/pharmacodynamics (PD) target considered. In all of these studies, the variability of antibiotic doses administered is striking; this, on the one hand, reflects the uncertainty of the clinician on how RRT may affect antibiotic concentrations but, on the other hand, adds a level of complexity when it comes to analyzing target attainment in different studies. Furthermore, the intervals between start of antibiotic therapy and sampling as well as RRT initiation and sampling are additional confounders when target attainment is analyzed in these studies. In a systematic review, Vaara and colleagues [6] found the overall quality of studies investigating the effect of RRT moderate, as the many important RRT parameters were often lacking.

In this issue of *Critical Care*, Beumier and colleagues [1] report that standard, unadjusted dosing results in very high concentrations in about half of the patients. Although the study includes a large number of patients and reflects clinical practice in their intensive care unit, there may have been a selection bias with only patients at risk selected for therapeutic drug monitoring (TDM). Also, the timing was variable, and it is not clear whether patients that were treated with antibiotics long before RRT was initiated had an increased risk of overdosing as it was defined in this study.

With these new data, drawing firm conclusions regarding antibiotic dosing during RRT remains difficult. However, it can be concluded that higher-than-standard dosing in this patient population is probably unwarranted. From previous studies, it seems that underdosing is more common during the first days of treatment and that overdosing may occur later (48 hours or later) when doses are not adjusted. This is probably due to the fact that factors other than RRT determine the concentrations in the first 24 to 48 hours of therapy, such as the enhanced volume of distribution, and elimination determines the subsequent concentrations. Furthermore, it should be kept in mind that the susceptibility of the microorganism is equally important; target attainment is in danger only when borderline susceptible microorganisms with higher-than-average MIC are involved.

Clearly, more research using well-designed studies with prospective data collection is needed. Moreover, the impact of other RRT strategies such as intermittent hemodialysis and sustained low-efficiency daily dialysis needs our urgent attention. In the meantime, for patients undergoing CRRT, optimizing antibiotic therapy is as essential as in other patients. Though rarely suggested in CRRT, prolonged and extended infusion may be sufficient to overcome underdosing in many. In difficult cases, TDM - when available - could be a solution, although many questions surrounding the application of TDM remain unanswered. Most importantly, it is not clear what PK/PD target should be aimed for to treat infections during critical illness, but TDM could at least detect the extremes of concentrations and allow dose adaptation in case of severe under- and overdosing.

## Abbreviations

CRRT: Continuous renal replacement therapy; MIC: Minimum inhibitory concentration; PD: Pharmacodynamics; PK: Pharmacokinetics; RRT: Renal replacement therapy; TDM: Therapeutic drug monitoring.

## Competing interests

The authors declare that they have no competing interests.
